# Peri-partum cardiomyopathy in a pregnant woman at term revealed by acute pulmonary edema: what to do in front this catastrophic situation?

**DOI:** 10.11604/pamj.2014.18.29.3782

**Published:** 2014-05-08

**Authors:** Hatim El ghadbane Abdedaim, Zine el abidine Benali, Driss Omari, Drissi Mohammed, Balkhi Hicham, Haimeur Charki

**Affiliations:** 1 Department of Anesthesiology & Intensive Care, Military Hospital Mohammed V, University Mohammed V Souissi, Rabat, Morocco; 2 Department of Anesthesiology & Intensive Care, CHP Eddarak, Berkane, Morocco; 3 Department of Internal Medicine and Cardiovascular Diseases, CHP Eddarak, Berkane, Morocco

**Keywords:** Peri-partum, cardiomyopathy, acute pulmonary edema

## Abstract

Peripartum Cardiomyopathy is insufficient congestive heart occurring in the last month of pregnancy and 5 months after delivery, in the absence of preexisting heart disease and identified etiology. This heart disease is associated with echocardiography systolic dysfunction and left ventricular dilatation. Its incidence ranges from 1/3000 to 1/15000, depending on the region, including much higher in some African countries, it particularly concern women over 30 years, multiparous and multiple pregnancies. The pathogenesis remains unclear, the prognosis is closely related to the complete recovery of cardiac function. We report through the clinical case of a woman aged 33 years admitted to the ICU for acute pulmonary edema of sudden onset of a term pregnancy and what to do before this critical situation

## Introduction

Peripartum cardiomyopathy (PC) is a heart disease last month and postpartum, with rare and serious complications, its diagnosis is based on well-defined universal criteria based on clinical and echocardiography. We report through this clinical case of a patient without cardiac history, nulliparous, aged 33 years, admitted in ICU whose master symptom is acute pulmonary edema

## Patient and observation

**Figure 1:**
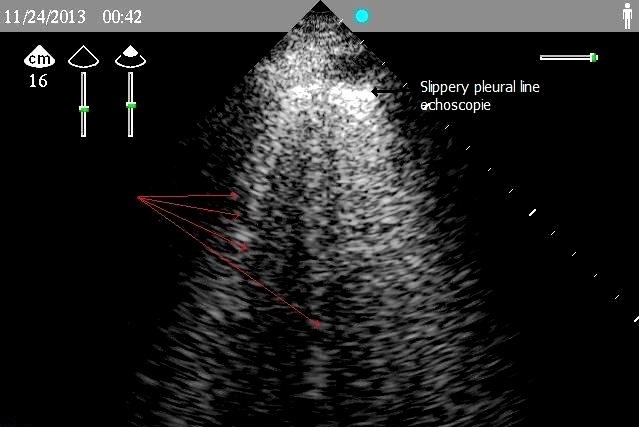
2D lung ultrasound showing the presence of several vertical lines B in favor of acute pulmonary edema (red arrows)

**Figure 2:**
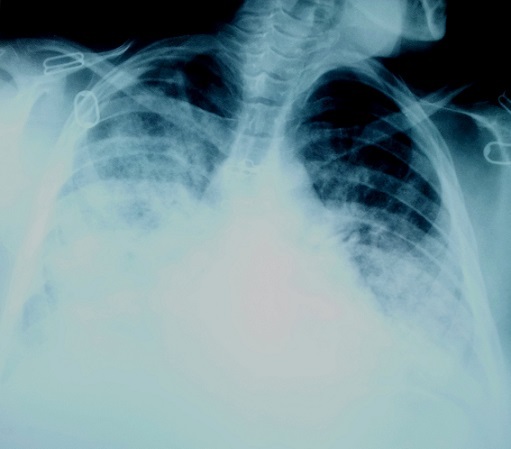
Chest radiograph showing bilateral opacities in favor of the presence of fluid in the alveoli

**Figure 3:**
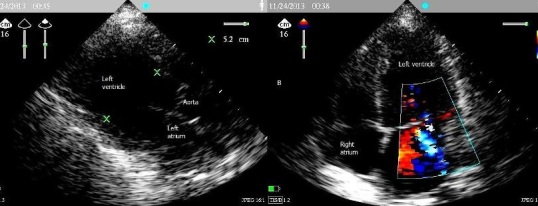
A) 2D echocardiography parasternal long axis showing a left ventricular dilatation in diastole with 3.25 cm/m^2^ (calculated body surface area according to the Mosteller formula: S = √ (L ×M /3600); S is the body surface area in m^2^, L is the size in cm, M is the mass in kg); B) apical four-chamber view with color Doppler showing mitral regurgitation grade I to II

A woman aged 33 years, nulliparous, pregnant 38 weeks gestation, normotensive, admitted to the maternity ward for irregular uterine contractions, with history as a nocturnal dry cough appears a week ago with dyspnea NYHA stage II, the standard laboratory tests unremarkable, examination gynecology - obstetrics showed a long cervix closed, six hours after the patient had suddenly unexplained acute dyspnea, the patient was admitted directly to ICU whose examination showed: buccal and cyanosis extremities, dyspnea NYHA stage III, saturation oxygen for 7 liter / min was 85%, blood pressure was 130/80 mm Hg, a temperature of 37 ° c, the cardiopulmonary examination revealed crackling rales in both lung fields levels, tachycardia 96 beats / min without obvious breath, ECG showed: sinus tachycardia, without signs of pulmonary embolism or of myocardial infarction. the bilateral lung ultrasound showed B vertical lines far beyond 3 lines on the ultrasound screen (Figure 1) in favor of acute pulmonary edema, chest radiograph confirms the hypothesis (Figure 2), the echocardiogram showed (Figure 3): a global hypo kinesis with ejection fraction (EF) 40%, a left ventricular dilatation with diastolic dimension: 3.25 cm/m^2^(body surface area for this patient is 1.6), no right or left atrial dilatation, mitral insufficiency grade I to II, pulmonary arterial hypertension moderate, right ventricular contractility was normal, dry pericardium. Flash a fetal echocardiogram showed bradycardia to 90 beats / min, an indication of emergency caesarean section was landed without loss of time to rescue mother and fetal. patient was placed under furosemide 1 mg / kg in 200 ml of saline serum to pass 5 min, low dose dobutamine 5 gamma / kg / min, who showed echo cardiographic left ventricular positive response in favor of contractile reserves, Premedication consisted of 200 mg cimetidine, hydroxyzine, prophylactic antibiotic therapy based on beta lactam. Throughout the procedure, blood pressure, oxygen saturation, heart rate, urine output, and ECG were Monitored. Spinal anesthesia with isobaric bupivacaine 8 mg of 0.5%, and half Assisi position just after, with nasal oxygen 10 liter / min. The surgeon was advised to expedite its action, extraction of a male baby APGAR score: 8/10 with a weight of 3.5 kg. Anticoagulants curative dose based enoxaparin subcutaneously was established at the tenth hour postoperative, multimodal analgesia, diuretics, filling echo guided, antibiotic prophylaxis for 48 hours. Inhibitors of angiotensin-converting enzyme and the specific beta-blockers started the third day after the stoppage of dobutamine. After seven days, Echocardiography showed ejection fraction 50%.the patient was transferred on the eleventh day in maternity unit with cardiac monitoring.

## Discussion

Peri partum cardiomyopathy (PC) is an idiopathic dilated cardiomyopathy that occurs during the last stage of pregnancy or after childbirth. It was described for the first time in 1870 when Virchow and Porak reported signs of myocardial degeneration at autopsy dead in the postnatal period [1]. PC occurs during pregnancy in 3000 to 15000. Its incidence appears to be higher among Africans, but all races are affected. Women at particular risk are: older women, multiparous, who received tocolytic treatment, which were suffering from preeclampsia or who gave birth to twins [2]. Its pathophysiology remains poorly understood, is probably multifactorial. Pregnancy is a state of latent heart failure and pro -inflammatory state which can graft a dysimmunity, an infectious state, but two things seem still more decisive: falling estrogen and prolactin production. Hence the hypothesis, recent that peripartum cardiomyopathy occurs on an imbalance between estrogen and prolactin [3].

The PC is defined on the basis of four criteria, adapted from the work of Demakis et al: Occurrence of heart failure in the last month of pregnancy or 5 months after, heart failure did not cause identifiable, absence of recognizable heart disease before the last month of pregnancy, left ventricular dysfunction demonstrated by classic criteria for echocardiography: ejection fraction 2.7 cm/m^2^ body surface area. Our patient met all clinical and echocardiography criteria. The main differential diagnoses to be eliminated are: pulmonary embolism, acute pneumonia, acute coronary syndrome, decompensation of a pre-existing heart disease, cardiac tamponade and acute aortic syndrome. Clinical, ECG, biology and ultrasound provide redress diagnosis. Echocardiographic diagnosis of gravity is represented by: Severity of dilatation of the left cavities more than 60 mm, Severity of impaired left ventricular function (EF 3,4]. Treatment during the acute phase in pregnant women at term includes: half sitting position, oxygen high flow, diuretics in case of congestive heart failure, dobutamine if left ventricular dysfunction confirmed by echocardiography, a Caesarean section under spinal anesthesia is indicated for the emergency rescue for maternal and fetal, the cardiovascular benefit of early delivery is the most important element, as in our patient. In postpartum: anticoagulants curative dose is indicated, inhibitors of angiotensin-converting enzyme and beta-blockers. If not, the circulatory support finds its place in case of refractory cardiogenic shock [3, 5–7].

The risk of recurrence of the PC in a subsequent pregnancy is related to the degree of left ventricular dysfunction underlying. However, even if left ventricular function is normalized 6 months after delivery, there remains a risk of heart failure during a future pregnancy. The prognosis depends mainly on the normalization of ventricular function six months after delivery [8]. The estimated mortality rate associated with PC in the United States is 6% to 10%, death usually occurs within 30 days but has occurred later as well [9, 10]. What we required a particularly close cardiac monitoring during the first months.

## Conclusion

Peripartum cardiomyopathy is a serious heart disease by complications, unknown etiology and that requires taking prompt and adequate multidisciplinary management, echocardiography is a diagnostic tool and prognosis. Should be recommended to women who did not normalize ventricular function avoid another pregnancy. it must be kept in mind that the risk of recurrence in future pregnancies persists even after complete recovery of cardiac function, which requires us a cardiac monitoring for each pregnancy up to the menopause.
